# Filtering High-Dimensional Methylation Marks With Extremely Small Sample Size: An Application to Gastric Cancer Data

**DOI:** 10.3389/fgene.2021.705708

**Published:** 2021-07-12

**Authors:** Xin Chen, Qingrun Zhang, Thierry Chekouo

**Affiliations:** ^1^Department of Mathematics and Statistics, University of Calgary, Calgary, AB, Canada; ^2^Alberta Children's Hospital Research Institute, University of Calgary, Calgary, AB, Canada; ^3^Department of Biochemistry and Molecular Biology, University of Calgary, Calgary, AB, Canada

**Keywords:** DNA methylation, BACkPAy, LIMMA, Bayesian model, gastric cancer

## Abstract

DNA methylations in critical regions are highly involved in cancer pathogenesis and drug response. However, to identify causal methylations out of a large number of potential polymorphic DNA methylation sites is challenging. This high-dimensional data brings two obstacles: first, many established statistical models are not scalable to so many features; second, multiple-test and overfitting become serious. To this end, a method to quickly filter candidate sites to narrow down targets for downstream analyses is urgently needed. BACkPAy is a pre-screening Bayesian approach to detect biological meaningful patterns of potential differential methylation levels with small sample size. BACkPAy prioritizes potentially important biomarkers by the Bayesian false discovery rate (FDR) approach. It filters non-informative sites (i.e., non-differential) with flat methylation pattern levels across experimental conditions. In this work, we applied BACkPAy to a genome-wide methylation dataset with three tissue types and each type contains three gastric cancer samples. We also applied LIMMA (Linear Models for Microarray and RNA-Seq Data) to compare its results with what we achieved by BACkPAy. Then, Cox proportional hazards regression models were utilized to visualize prognostics significant markers with The Cancer Genome Atlas (TCGA) data for survival analysis. Using BACkPAy, we identified eight biological meaningful patterns/groups of differential probes from the DNA methylation dataset. Using TCGA data, we also identified five prognostic genes (i.e., predictive to the progression of gastric cancer) that contain some differential methylation probes, whereas no significant results was identified using the Benjamin-Hochberg FDR in LIMMA. We showed the importance of using BACkPAy for the analysis of DNA methylation data with extremely small sample size in gastric cancer. We revealed that RDH13, CLDN11, TMTC1, UCHL1, and FOXP2 can serve as predictive biomarkers for gastric cancer treatment and the promoter methylation level of these five genes in serum could have prognostic and diagnostic functions in gastric cancer patients.

## 1. Introduction

**Figure 1:**
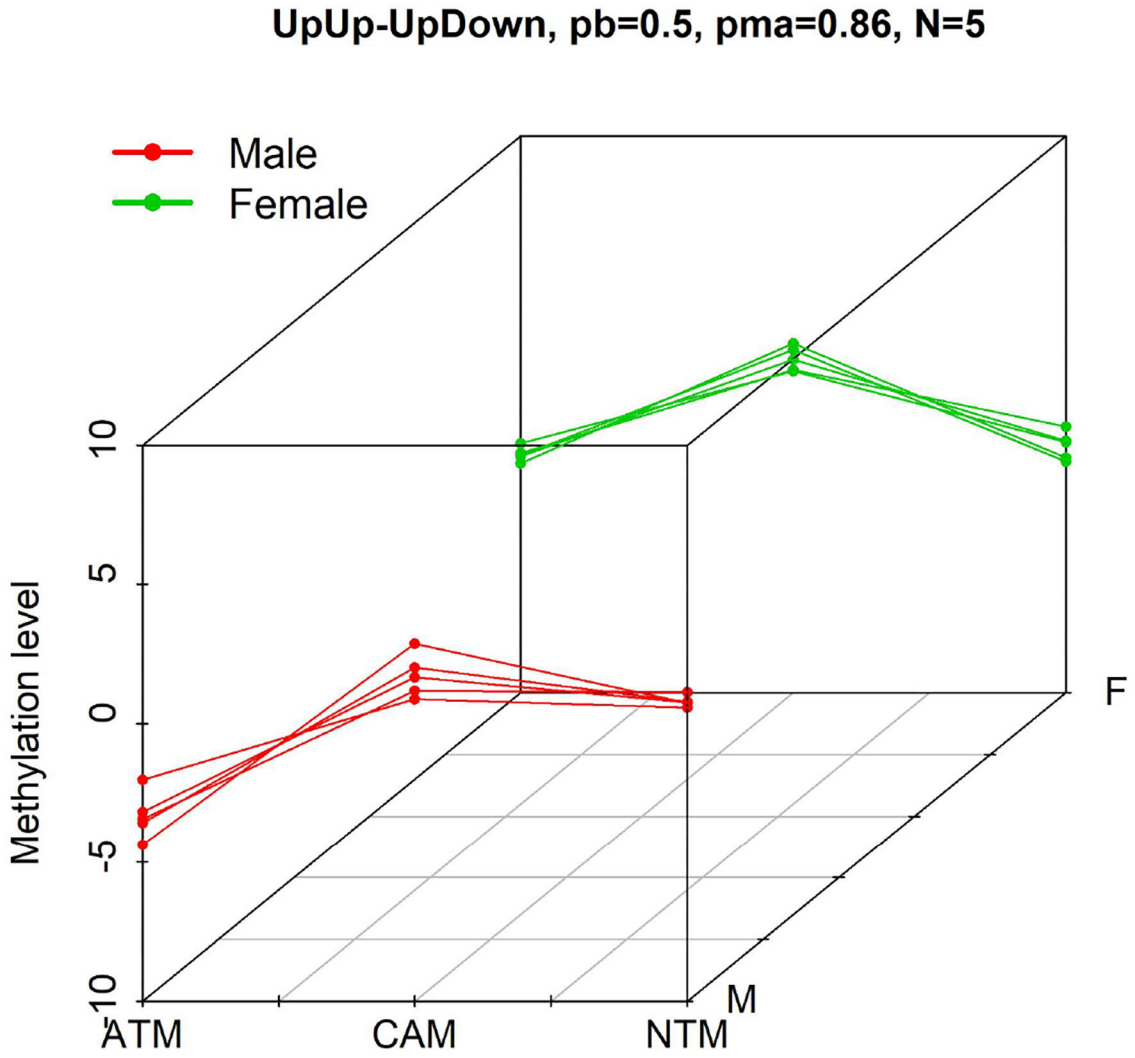
Example of probes with differential methylation level pattern, UpUp-UpDown, among three groups when comparing male (red—UpUp trajectory in this example) to female (green—UpDown trajectory in this example) samples. “*N* = 5” means that five differential methylation probes from GSE97686 dataset belong to this pattern with probability larger than pb = 0.5. pma = 0.86 means that the maximum probability to belong to this pattern is 0.86.

DNA methylation is a biochemical process of adding a methyl group at the 5' carbon of the cytosine ring in a nucleotide (Du et al., 2010; Li et al., 2015). It is an epigenetic modification in which chemicals tag DNA and regulate gene expressions. Promoter DNA methylation is associated with genes silencing, which contributes to the development of diseases, especially cancers (Ma et al., 2013). An active research field is to detect probes associated with differential methylation levels under contrasting conditions (e.g., sex and tissue types). However, the dimensionality of the problem makes it much harder. The number of features (probes) in methylation dataset is typically at least on the order of several thousand, whereas the number of samples may be few, presenting challenges in multiple hypothesis testing as well as overfitting. In this manuscript, we are interested in identifying or filtering groups of potential probes that show significant methylation level differences (and similar patterns) among experimental conditions while accounting for another demographic factor (e.g., sex). In particular, using a DNA methylation dataset in gastric cancer with extremely small sample size (e.g., in cell line experiments), we would like to analyse differential methylation probes among experimental groups for both male and female (see Figure 1 for instance).

A detailed review of current methods used for differential methylation analysis can be found in literature (Wilhelm-Benartzi et al., 2013). Some methods along with some software and R packages have been developed to detect probe-wise or locus-specific methylation differences between specific groups (Wettenhall and Smyth, 2004; Zackay and Steinhoff, 2010; Barfield et al., 2012; Kilaru et al., 2012; Wang et al., 2012; Hansen and Aryee, 2013). For instance, the package *MethVisual* can be applied to test whether each CpG site has independent membership between two groups using Fisher's exact test (Zackay and Steinhoff, 2010). The *Minfi* R package uses linear regression and an *F*-test to test for a univariate association between methylation level of single loci and phenotypes (Hansen and Aryee, 2013). Bayesian Gaussian models can also be used based on methylation levels with *M*-values and they rely heavily on the Gaussianity assumption (Zhuang et al., 2012).

The aforementioned methods as well as many traditional statistical methods such as *t*-test/*F*-test require moderate or large sample sizes. When sample sizes are extremely small (e.g., in cell line experiment), these methods tend to not control the type-1 error rate accurately, which means they may over-reject the null hypothesis. Therefore, the results of hypothesis testing have low statistical power and more advanced statistical methods should be taken into consideration. Some exact techniques (i.e., procedures that rely on the exact distribution of a test statistic) work with small sample sizes, but they typically rely on strict model assumptions that can hardly be verified—at least in more complex models (Konietschke et al., 2021). Therefore, it is quite hard to obtain meaningful results by applying existing statistical methods when comparing methylation levels among different groups with extremely small sample size in each group. In our study, GSE97686 dataset contains DNA methylation levels among three tissue types, and there are just three samples in each tissue type. For example, the ATM group (patient-matched adjacent tissue myofibroblasts) defined in Najgebauer et al. (2019) only contains one female and two male samples. In this case, LIMMA (Linear Models for Microarray Data) may be a suitable choice for DNA methylation data, which is used to assess differential expression in the context of multifactor designed experiments and has features making the analyses stable even for data with small sizes. However, LIMMA does not provide clusters of differential features that may ease their biological interpretations.

To this end, our novel method BACkPAy (BAyesian mixture model for identifying Clusters of features (e.g., proteins) with similar “pre-defined” expression PAtterns) is a more suitable choice over LIMMA, which is developed to identify features (probes) that are differentially expressed in varying conditions for downstream analyses (Chekouo et al., 2020). More specifically, the method relies on a constrained Bayesian mixture model for clustering that groups omics features (e.g., proteins, methylation probes) into biologically meaningful clusters. Patterns of differentially features in varying conditions are obtained as combinations of these clusters. In particular, by applying BACkPAy to our methylation dataset, our aim is to group methylation probes based on their methylation profile over the three different tissue types for male and female samples. Unlike LIMMA, BACkPAy allows to filter potentially significant probes by Bayesian FDR method while obtaining several biologically meaningful patterns/groups (e.g, UpUp-UpDown, UpDown-UpUp) of probes. Additionally, in Chekouo et al. (2020), it has been shown that BACkPAy achieved better performance than competing methods (LIMMA included) across different sample sizes. In particular, when the sample size is one in each experimental group, other methods can not be applied, but BACkPAy achieved satisfactory results.

In this paper, we filtered significant CpG sites with *p* < 0.05 by ANOVA (Analysis of variance) from GSE97686 dataset, which contains methylation levels of 424383 probes in gastric cancer. Then, 15,504 probes passing this critical value were used to detect the methylation differences among three groups comparing male to female samples by two statistical methods: LIMMA (Linear Models for Microarray Data) and our innovative Bayesian method (BACkPAy). To identify significant prognostics biomarker genes for gastric cancer, we applied a Cox model using the mRNA expression of the genes that map to the Illumina IDs of probes with differential methylation level filtered by BACkPAy. Gene expression data was extracted from TCGA-STAD (The Cancer Genome Atlas Stomach Adenocarcinom) dataset including 238 observations with 26,540 mRNA markers. Five significant genes with adjusted *p* < 0.05 were selected. In order to further compare the changes of methylation levels and gene expression levels among groups, CAM (primary gastric cancer-associated myofibroblasts), ATM (patient-matched adjacent tissue myofibroblasts) and NTM (unrelated normal tissue myofibroblast), the methylation data and gene expression data of these five genes was collected from GSE97686 and GSE107161, respectively (gene expression data with 47,312 probes from gastric stromal myofibroblast) in **Figures 9**–**13**.

In section 2, we introduced statistical methods in details. In section 3, we compared the results of two methods through methylation data of gastric cancer. The filtered significant probes by BACkPAy were separated into patterns with differential methylation levels. We also detected significant genes with prognostic function in gastric cancer by Cox model based on TCGA data relevant to potential significant probes filtered by BACkPAy and summarized some methylation biomarkers in different gastric groups (ATM, CAM, and NTM). By the analysis of changes in DNA methylation and corresponding RNA gene expression, the effects of hypomethylation or hypermethylation among different gastric groups in terms of these biomarkers have been discussed. Finally, we concluded with a brief discussion in section 4.

## 2. Materials and Methods

### 2.1. Datasets

In this work, we used a dataset with very small sample size and a large number of features, which is available from the National Center for Biotechnology Information (NCBI) Gene Expression Omnibus (GEO) public functional genomics data repository with GEO number GSE97686 and GSE107161 (Najgebauer et al., 2019). They are originated from the Illumina Infinium HumanMethylation450 BeadChip arrays with 424,383 probes and obtained from nine blood samples with gastric stromal myofibroblasts. Sex and tissue type are treated as experimental and independent variables, respectively. There are three different tissue types: primary gastric cancer-associated myofibroblasts (CAM), patient-matched adjacent tissue myofibroblasts (ATM), and unrelated normal tissue myofibroblasts (NTM). Each tissue type contains three samples. Before analyzing methylation data, we pre-processed the original β values in GSE97686 by logit transformation to get *M*-values that are more statistically valid for the differential analysis of methylation levels (Du et al., 2010). Similarly, GSE107161 gene expression data contains total RNA with 47,312 genes obtained from gastric stromal myofibroblasts, including three CAMs, three ATMs, and three NTMs, and hybridized to the Illumina HumanHT-12v4 Expression BeadChip.

Some preliminary processing steps have been applied to the two datasets: (1) we remove genes with missing expression values for GSE107161; (2) and applied ANOVA to filter probes/genes with significant differences among tissue types (*P*-value < 0.05). BACkPAy and LIMMA were applied to the remaining 15,504 probes and 738 genes for the DNA methylation and gene expression datasets, respectively.

In this manuscript, we also used the survival data of gastric cancer from TCGA-STAD dataset, The Cancer Genome Atlas Stomach Adenocarcinoma. TCGA-STAD level-3 mRNA expression data contains 238 sample subjects with 26,540 mRNA markers. The level-3 mRNA expression data has been quantified by mRNA Analysis Pipeline (Grossman et al., 2016).

### 2.2. Quantitative Method of DNA Methylation

In DNA methylation analysis, β value is a quantitative indicator of the methylation level. The formula is shown below

(1) $$\begin{array}{r} \beta =\frac{Max\left(M,0\right)}{Max\left(M,0\right)+Max\left(U,0\right)+100}, \end{array}$$

where *Max*(*M*, 0) is the intensity of methylated allele, while *Max*(*U*, 0) is the intensity of unmethylated allele (Du et al., 2010; Li et al., 2015). β value varies between 0 and 1, which represents the degree of DNA methylation in a sample. Generally, “zero” indicates there is no DNA methylation in CpG sites of the sample; “one” means that the focal CpG site in all the cells of the sample is methylated. Additionally, we used 0.2 and 0.8 as the thresholds of hypomethylation and hypermethylation. Alternatively, a β value could be transformed to a *M*-value by the following formula

(2) $$\begin{array}{r} M=log_{2}\frac{\beta }{1-\beta }. \end{array}$$

We can see the *M*-value and β-value are related through a log-2 ratio transformation. However, the range of *M*-value is from −inf to +inf, which is larger than β value. In this case, “zero” indicates the sample is half-methylated. And positive values mean a methylation rate >50% while negative values suggest a methylation rate <50% (Du et al., 2010; Li et al., 2015).

### 2.3. BACkPAy

BACkPAy is a recently released software that implements a Monte Carlo Markov chain (MCMC) algorithm for the detection of omics patterns (Chekouo et al., 2020). The software is available via this link: https://github.com/chekouo/BAckPAy. BACkPAy is one of the few statistical methods that is able to detect omics patterns while identifying differential omics features between experimental conditions when sample sizes are extremely small. Here, we provide a general overview of the method. Additional details on the BACkPAy architecture as well as on the software can be found in Chekouo et al. (2020).

**Figure 2:**
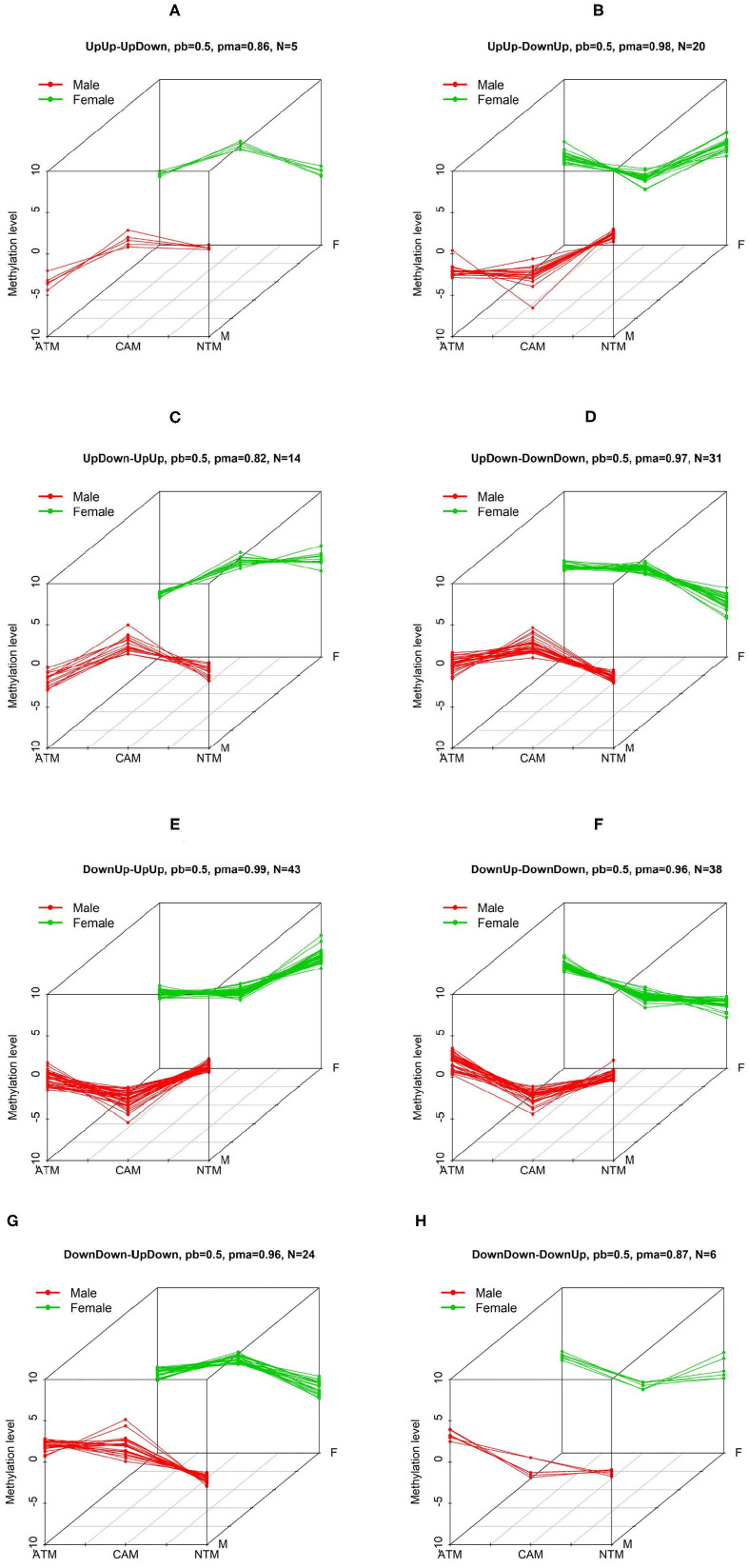
The first 6 three-dimensional patterns of differential methylation levels. Two other patterns are shown below. Three-dimensional patterns of differential methylation levels. Eight patterns **(A–H)** with differential methylation levels between three groups (CAM, ATM, and NTM) when comparing male to female samples were identified by BACkPAy. Each group contains three samples (one male and two female samples or two male and one female samples). In each panel, *N* represents the number of differential methylation level probes belonging to one specific pattern with probability larger than pb = 0.5. pma is the maximum probability to belong to the corresponding pattern.

Overall, the methylation data is encoded as **Y** = (**y**_*s*_, *j* = 1, …, *p*; *s* = 1, 2), where **y**_**js**_ = (*y*_*js*1_, *y*_*js*2_, …, *y_jsn_s__* is a vector in which each element *y*_*jsi*_ represents the *M*-value of probe *j* in sample *i* = 1, …, *n*_*s*_ of type *s*, *s* = 1 if the sample is male and *s* = 2 if female. Then, we assume that *y*_*js*_ comes from a mixture of a finite number *H* of components. Given the *h*'th component (cluster) of the mixture, the model is written as

(3) $$\begin{array}{r} y_{jsi}=a_{jh}+x_{si1}\beta_{h1}+x_{si2}\beta_{h2}+\epsilon_{jsih},\;\epsilon_{jsih}\sim Normal\left(0,\sigma_{h}^{2}\right), \end{array}$$

where *a*_*jh*_ is the probe-specific random effect of probe *j* within cluster *h*, *x*_*si*1_ = 1 (i.e., *k* = 1) if sample *i* of type *s* (male or female) is from ATM group, and 0 otherwise; and *x*_*si*2_ = 1 (i.e., *k* = 2) if sample *i* of type *s* is from NTM group, and 0 otherwise. Finally, (β_*h*1_, β_*h*2_) is the vector of slopes that measures the tissue type group effects. Through prior distributions of the slopes, we defined *H* = 9 clusters with respect to the signs of the coefficients β_*sh*1_ and β_*sh*2_. For instance, cluster 1 (DownUp) is characterized by features with decreasing methylation level from ATM group to CAM group and increasing methylation level from CAM group to NTM group (β_11_ > 0 and β_12_ > 0), and cluster 2 (FlatUp) is characterized by features with constant methylation level from ATM to CAM group and increasing methylation level from CAM group to NTM (β_21_ = 0 and β_22_ > 0), etc. For instance, pattern of features DownUp-FlatUp is a group of probes that belong to clusters DownUp and FlatUp for male and female samples, respectively. To implement BACkPAy, we set the hyperparameters of the parameters τ_*sh*_'s which help to constrain the regression coefficients in Equation (3) and allow to make a clear distinction between the clusters (e.g., UpUp, UpDown). Formally, τ_*sh*_ follows a Gamma distribution with parameters *a*_τ_ = 7 and *b*_τ_ = 5 (i.e., prior mean of τ_*sh*_ is 1.4) when β_*sh*_ ≠ 0 for both DNA methylation and gene expression data. For the estimation in BACkPAy, the total number of MCMC draws was 30,000 iterations, with 10,000 discarded as burn-in. Further, we plotted patterns of features with inclusion probabilities larger than 0.5. The maximum of those inclusion probabilities for each pattern was also shown on the graph (see Figure 2 for instance).

### 2.4. LIMMA

LIMMA is a popular R/Bioconductor software package using linear models with robust hyperparameter estimation to assess differential methylation levels in several (more than 2) groups. We fitted a linear model using lmFit function in R to estimate *β*_***j***_, the differences among three groups for the *j*'th probe. Let $y_{j}^{T}=\left(y_{j_{1}},...,y_{j_{n}}\right)$ define the DNA methylation level (*M*-value) for three groups with *n* = *n*_1_+*n*_2_+*n*_3_ for the *j*'th probe. The expected value of ***y***_*j*_ is defined as *E*(***y***_*j*_) = ***Xα***_*j*_, where ***X*** is a design matrix providing a representation of the different DNA methylation targets that have been hybridized to the arrays, ***α***_*j*_ is a vector of coefficients. $\beta_{j}=C^{T}\alpha_{j}$, where ***C*** is a contrast matrix. Thus, the null hypothesis for testing the DNA methylation differences between male and female in each group is *H*_0_:β_*jt*_ = 0 for probe *j* = 1, …, *n* and *t* = 1, 2, 3 representing three groups of tissue type. The test statistic for testing *H*_0_ is the moderated t-statistic, based on a Bayesian approach, defined by

(4) $$\begin{array}{r} {\tilde{t}}_{jt}=\frac{{\hat{\beta }}_{jt}}{{\tilde{s}}_{j}\sqrt{v_{jt}}}. \end{array}$$

The *p*-value for testing *H*_0_ is calculated from the *t* distribution with *d*_*j*_ + *d*_0_ degrees of freedom. More information on $\tilde{s_{j}},v_{jt},d_{j}$ and *d*_0_ could be found in Phipson et al. (2016). If the *p*-value of ${\tilde{t}}_{jt}$ is <0.05, we could reject the null hypothesis *H*_0_, i.e., there is a significant difference between male and female for probe *j* in group *t*. Conversely, If *p*-value is larger than 0.05, that means there is no difference between male and female for probe *j* in group *t*.

To account for the multiplicity of tests, we adjusted the *p*-values obtained from LIMMA using the False discovery rate (FDR) approach of Benjamini and Hochberg (1995). More details about the procedure are provided in the Supplementary Material (section 2). Storey (2003) introduced a modified version of the FDR that allows to define *q-values* which is a natural Bayesian posterior *p*-value. BACkPAy uses *q*-values to detect differential features and provides *q*-values of each feature with respect to each pattern/group.

To identify prognostic markers, we used Cox regression models defined by the hazard function

(5) $$\begin{array}{r} h\left(t\right)=h_{0}\left(t\right)\exp\left\{b_{1}X_{1}\right\} \end{array}$$

where *h*_0_(*t*) is the baseline hazard function at survival time *t*, the coefficient *b*_1_ measures the effect of variable *X*_1_ (e.g., gene expression) [see Supplementary Material (section 3) for more details].

## 3. Results

### 3.1. Differentially Probes/Genes Using BACkPAy and LIMMA

Using the GEO DNA methylation data, BACkPAy identified 11,834 (out of 15,504) potential differential probes (Bayesian *q*-value <0.05) while we got only 1,080 differential probes using LIMMA (*p*-value with F distribution <0.05). In addition, after adjusting the *p*-values obtained by LIMMA with the Benjamin-Hochberg approach (Benjamini and Hochberg, 1995), we were not able to find any significant probes (adjusted *p*-value <0.05). In BACkPAy, non-differentially probes belong to group FlatFlat-FlatFlat i.e., probes that do not have a significant change between tissue types for male and female samples. We note that in the presence of extremely small sample sizes (one or two samples per experimental condition), BACkPAy can be considered as a “pre-screen” method that screens out non-differential probes and keeps potential differential probes but not necessary important ones. On the other hand, for gene expression data (GSE107161), the number of potential differential genes filtered by BACkPAy is 10 out of 738, which is smaller than genes we got by LIMMA (34 out of 738). We were also not able to find any significant genes after adjusting the *p*-values for LIMMA. The 10 genes obtained by BACkPAy do not overlap with those filtered obtained from methylation dataset. All these results show that LIMMA has conservative statistical power when sample sizes are extremely small and it is hard to get any significant probes or genes after multiple testing correction. BACkPAy can filter potentially significant probes/genes by Bayesian *q*-values and also, can separate differential methylation probes into biologically meaningful patterns (see Figure 2).

### 3.2. Gene Expression Profiling With TCGA Data

We are interested in groups/patterns of differential methylation level probes between the three tissue types for male and female. We will focus on the eight patterns/groups as illustrated in Figure 2 using our GEO DNA methylation dataset. In each of the eight patterns, clusters for males is different from females (e.g., in Figure 2A), UpUp is cluster for male, and UpDown for female). We have then selected 181 significant probes with differential methylation level that are clustered into the eight patterns of interest. To further investigate probes of interest found using BACkPAy, we analyzed TCGA-STAD data that contains related gene expression data with approximately 238 cancer patients. We focused on mRNA expression and the overall survival time of those patients. From TCGA data, we extracted the mRNA expression of genes corresponding to 181 significant probes filtered by BACkPAy. We then fitted univariate Cox proportional hazard models for each gene (Supplementary Table 1) and five significant genes were filtered by adjusted *p*-value <0.05 (Bradburn et al., 2003). They are RDH13, CLDN11, TMTC1, UCHL1, and FOXP2.

**Figure 3:**
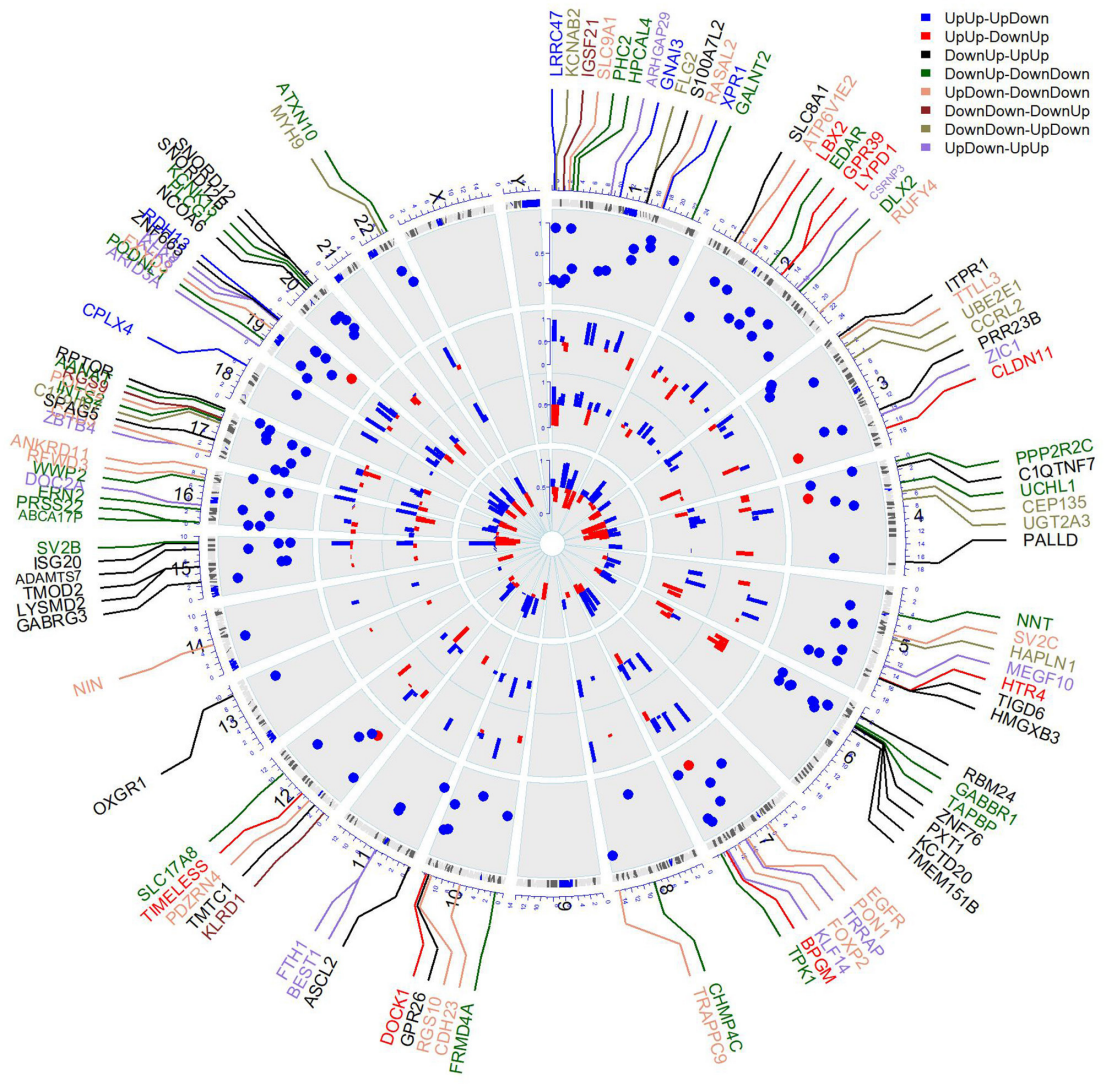
Circular plot of DNA methylation levels of significant genes in stromal myofibroblasts. The outermost ring represents human ideograms, i.e., genome positions by chromosomes. The black lines are cytobands. The labels outside the ring are the names of significant genes (probes) selected by BACkPAy. Different colors of these gene labels represent the differential methylation patterns significant genes belong to among ATM (adjacent tissue-derived myofibroblast), CAM (cancer-associated myofibroblast), and NTM (normal tissue-derived myofibroblast) groups comparing male to female samples; each tissue type contains three samples, Bayesian *q*-value ≤ 0.05. The first track shows the adjusted p-value of these prognostics genes with the survival data in TCGA package. (red: FDR *p*-value <0.05, blue: FDR *p*-value > 0.05). The second track, the third track and innermost track represent the DNA average methylation levels of corresponding CpG sites from significant genes among CAM, ATM, and NTM, respectively. (red: β value <0.2, hypermethylated loci, blue: β value>0.2). The complete list of genes (with colors) is available in the Supplementary Material (section 1).

To validate significant probes with differential methylation patterns mapping to the related genes, we generated the circular plot containing differential methylation probes and gene expression profiling among CAM, ATM, and NTM in Figure 3. In Figure 3, genes with the same color belong to the same pattern of interest (e.g., genes written in blue indicate that important methylation probes from those genes belong to pattern UpUp-UpDown). We can observe that genes in chromosomes 6, 15, and 20 have similar methylation patterns, DownUp-UpUp or DownUp-DownDown. That means genes in these chromosomes show identical methylation cluster (DownUp; decrease from ATM to CAM and increase from CAM to NTM) for males while they have two different clusters for females (UpUp and DownDown). On the other hand, the two genes, CHMP4C and TRAPPC9 in chromosome 8, show the same methylation cluster (DownDown) for females while the methylation cluster for males is totally different between the two genes (DownUp cluster in CHMP4C, UpDown cluster in TRAPPC9). This indicates that, for gastric cancer, Sex shows a strong effect on the methylation pattern in chromosomes 6, 8, 15, and 20.

Moreover, from the second, third and innermost tracks in Figure 3, there are 33 genes (KCNAB2, TTLL3, S100A7L2, etc.) showing hypomethylation or hypermethylation levels in CAM and ATM compared with non-cancer group NTM. It implies that hypomethylation or hypermethylation of genes is likely to have a predictive function for gastric cancer and different type of patients (male or female with different tissue type) would have differential hypomethylated or hypermethylated levels in details. As these genome regions show distinct DNA methylation patterns in myofibroblast populations, it is possible that these distinct DNA methylation patterns have significant influence on the growth or discovery of gastric tumors.

**Figure 4:**
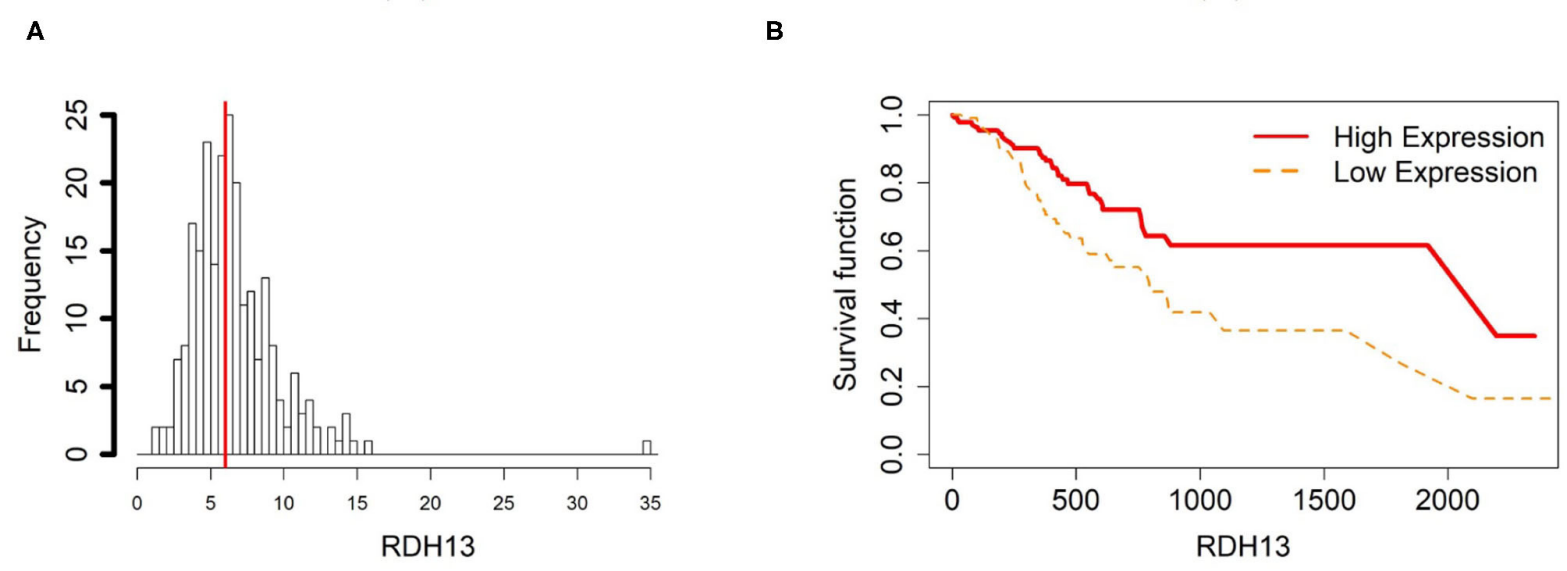
Survival analysis in RDH13. **(A)** Depicts the histogram of RDH13 gene expression. Based on TCGA-STAD dataset, we set 6 as the gene expression threshold to separate the 238 observations into two groups: low expression and high expression groups of samples. **(B)** Shows the estimated survival functions using a Cox proportional hazard regression analysis in RDH13 between the two groups; FDR *p*-value = 0.0325 indicates a significant survival time difference between the two groups.

**Figure 5:**
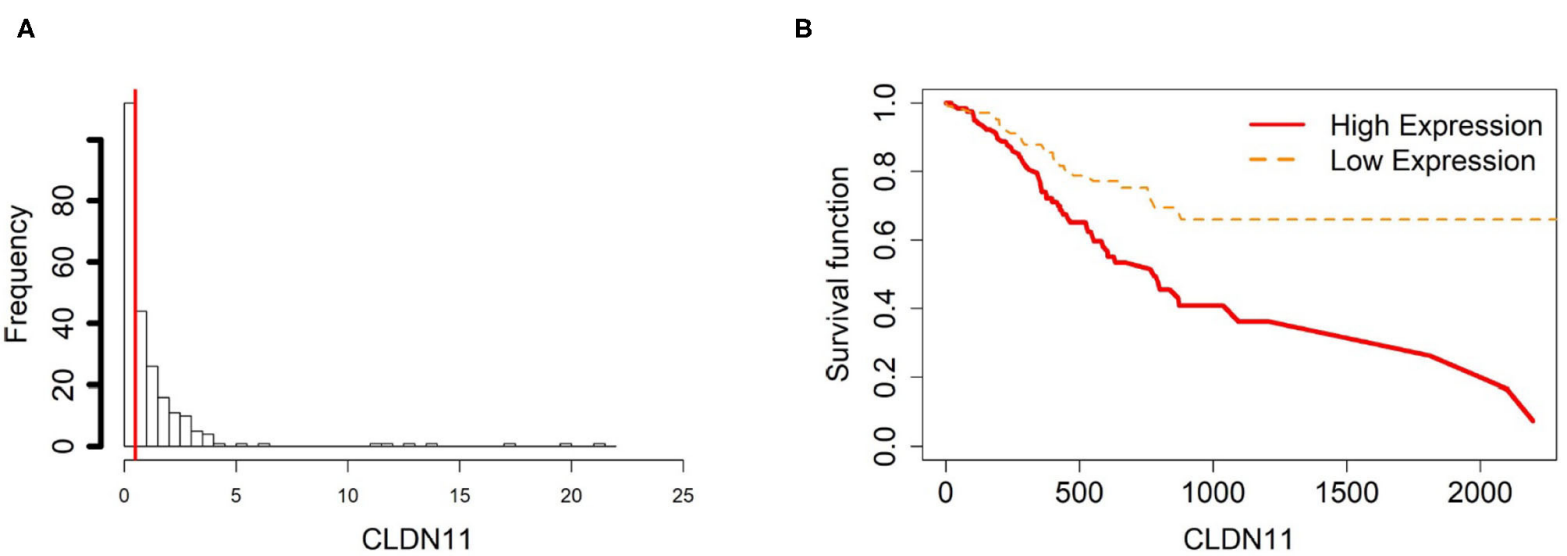
Survival analysis in CLDN11. **(A)** Describes the histogram of CLDN11 gene expression. Based on the CLDN11 data extracted from TCGA-STAD, we set 0.5 as the gene expression threshold to separate the 238 observations into two groups: low expression and high expression groups of samples. **(B)** Shows the estimated survival function plots using a Cox proportional hazard regression analysis in CLDN11 between the two groups; FDR *p*-value = 0.0325 indicates a significant survival time difference between the two groups.

**Figure 6:**
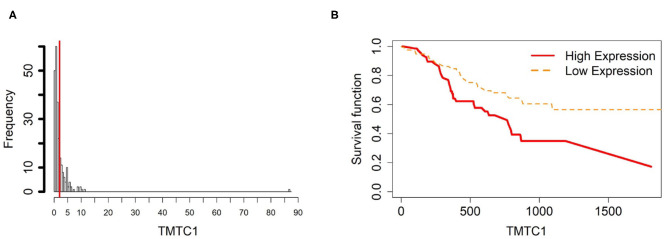
Survival analysis in TMTC1. **(A)** Depicts the histogram of TMTC1 gene expression. Based on the TMTC1 data from TCGA-STAD, we set 2 as the gene expression threshold to separate the 238 observations into two groups: low expression and high expression groups of samples. **(B)** Shows the estimated survival function plots using a Cox proportional hazard regression analysis between the two groups; FDR *p*-value = 0.0325 indicates a significant survival time difference between the two groups.

**Figure 7:**
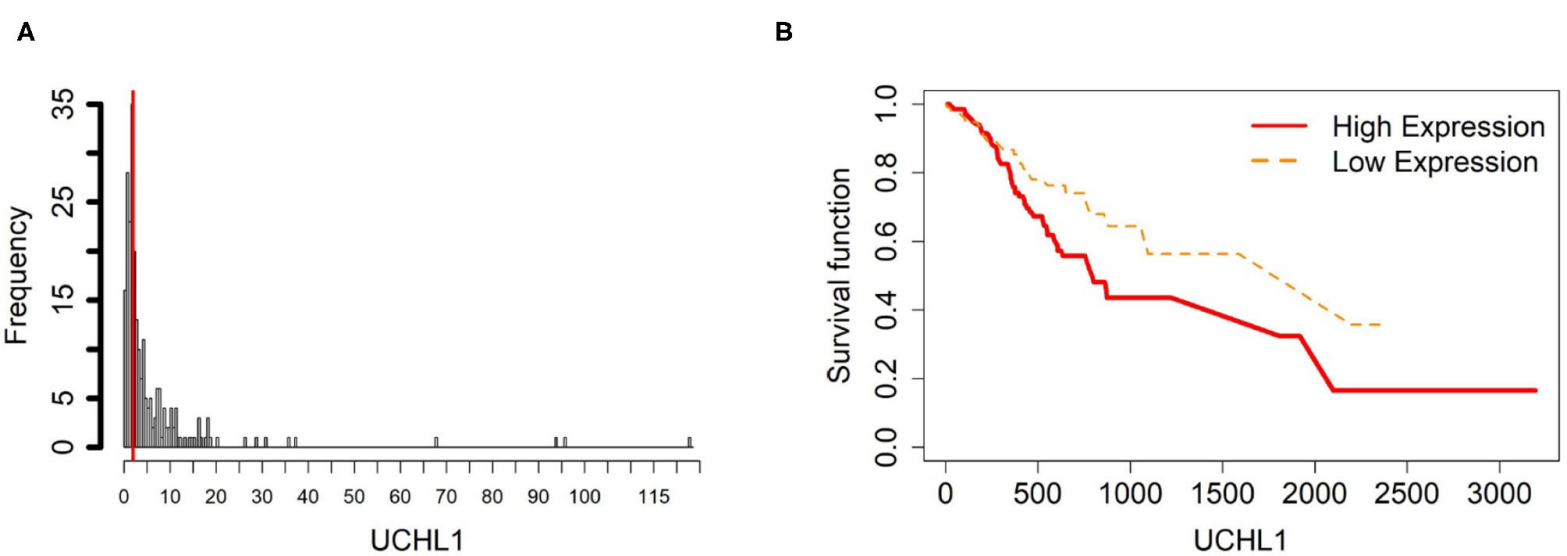
Survival analysis in UCHL1. **(A)** Depicts the histogram of UCHL1 gene expression. From TCGA-STAD dataset, we set 2 as the gene expression threshold to separate the 238 observations into two groups: low expression and high expression groups of samples. **(B)** Shows the estimated survival function plots using a Cox proportional hazard regression analysis between the two groups; FDR *p*-value = 0.0325 indicates a significant survival time difference between the two groups.

**Figure 8:**
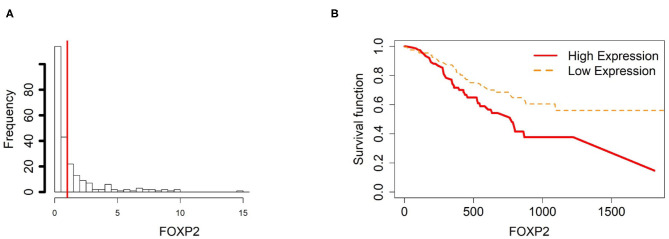
Survival analysis in FOXP2. **(A)** Depicts the histogram of FOXP2 gene expression. From TCGA-STAD dataset, we set 1 as the gene expression threshold to separate the 238 observations into two groups: low expression and high expression groups of samples. **(B)** Shows the estimated survival function plots using a Cox proportional hazard regression analysis between the two groups; FDR *p*-value = 0.0338 indicates a significant survival time difference between the two groups.

To further investigate the gene expression and function of the five prognostic genes in gastric cancer, (i) we created two groups of samples that have low and high mRNA expressions for each gene in Figures 4A–8A; and (ii) associated those two groups to the overall survival time of gastric cancer patients by Cox model. The corresponding survival curves of the two groups for each gene were generated in Figures 4B–8B. For gene RDH13, high expression is associated with increased survival time while for the other genes, CLDN11, TMTC1, UCHL1 and FOXP2, high expression is associated with decreased survival time.

### 3.3. Association of Genes RDH13, CLDN11, TMTC1, UCHL1, and FOXP2 With Previous Cancer Studies

Here, we identify and report the relationship between the five genes and previous cancer disease studies. It is known that RDH13 shares the greatest sequence similarity with RDH11, RDH12, and RDH14. And it has been studied that gene CLDN11 (claudin-11) has been shown to be silenced in gastric cancer via hypermethylation of its promoter region, and this hypermethylation is significantly correlated with downregulation of CLDN11 expression vs. normal tissues (Agarwal et al., 2009). It has also be shown that during the treatment of gastric cancer, differentially downregulated TMTC1 protein is identified in human gastric carcinoma cells (Mao et al., 2016), and TMTC1 is also found to be correlated with breast cancer at the functional level (Moccia et al., 2017).

Gene UCHL1, Ubiquitin C-terminal hydrolase-L1 (UCHL1) is a de-ubiquitinating enzyme. As pointed out in Gu et al. (2015), its function is controversial in different types of cancer diseases as it can be an oncogene (i.e., causes cancer) or a tumor suppressor. But, it has been reported that it has a higher positive expression rate in liver metastases from gastric cancer (Gu et al., 2015).

For gene FOXP2, several researches have also reported the roles of FOXP2 as a tumor suppressor in gastric cancer and other diseases like osteosarcoma and hepatocellular carcinoma. It is revealed that FOXP2 expression was associated with the regulation of microRNAs in cancer cells and FOXP2 could inhibit the growth of cancer cells by suppressing a series of cancer stem cell associated factors (Jia et al., 2016; Chen et al., 2018).

### 3.4. Methylation Profiling of Gastric Tumors Purified From Different Tissue Type and Different Sex in Each Tissue Type

We analyzed the methylation profiles of probes that map to five prognostic significant genes. We summarized the significant prognostic genes with adjusted *p*-values obtained by Cox model and the patterns of probes related to these genes by BACkPAy into Table 1. For gene RDH13 (or probe cg18743287), there is no significant methylation level difference between male and female in both CAM and ATM (Up-Up) while the difference can be seen in NTM (Up-Down). Similarly, methylation levels between male and female in NTM group are significantly different in gene UCHL1. In addition, the methylation levels between different sex in CAM group have significant differences in CLDN11, TMTC1, and FOXP2.

**Table 1:** Significant genes for diagnosis of gastric cancer by Cox model.

	**Probe**	**Gene name**	**Pattern**	**Adjusted *p*-value**	**Sign**
1	cg18743287	RDH13	UpUp-UpDown	0.0325	+
2	cg17078427	CLDN11	UpUp-DownUp	0.0325	−
3	cg05471616	TMTC1	DownUp-UpUp	0.0325	−
4	cg09921610	UCHL1	DownUp-DownDown	0.0325	−
5	cg20050108	FOXP2	UpDown-DownDown	0.0338	−

*The first four columns list prognostics probes filtered by BACkPAy, their related, corresponding groups/patterns of methylation levels and adjusted *p*-values calculated by Benjamin-Hochberg approach, respectively. The last column shows the effect of genes on gastric cancer survival time. + Indicates high gene expression associated with better survival time while − indicates high gene expression with poorer survival time*.

### 3.5. Promoter Hypomethylation Induces RDH13 Expression in Gastric Cancer

**Figure 9:**
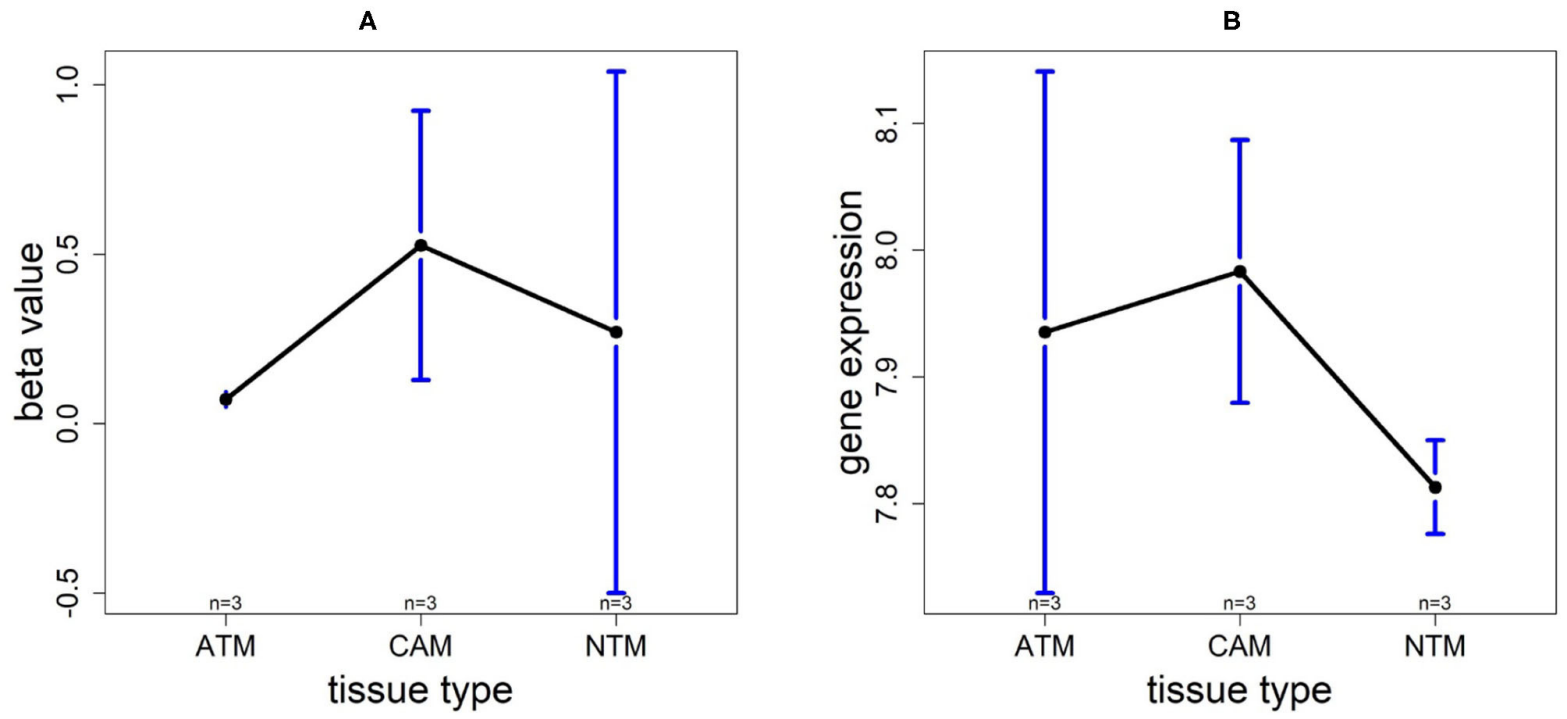
The overall methylation and gene expression levels of the RDH13 promoter region. **(A)** Averaged methylation level over the three samples of gene RDH13 among tissue types from GSE97686 dataset. **(B)** Averaged gene expression level over the three samples of gene RDH13 among tissue types from GSE107161 dataset. The blue bars represent 95% confidence intervals. Some error bars are large as the sample size is only 3 in each tissue type.

In order to compare the trends of gene expression levels and their corresponding methylation levels between tissue types for each of the 5 prognostics genes, we plotted the means of methylation levels and gene expressions in each tissue type based on GSE97686 and GSE107161 datasets, respectively (Figure 9). It reveals that the RDH13 promoter is hypomethylated in ATM tissue group, which means that its methylation level is lower in ATM than the other two tissue types (CAM and NTM). However, the gene expression among tissue types confirms that RDH13 is significantly upregulated in ATMs due to its hypomethylation compared to the other two tissue types, especially in normal tissues. Collectively, these results provide a strong indication that RDH13 expression may be induced by cancer-induced reprogramming, resulting in RDH13 promoter hypomethylation within gastric ATM group. That is, the hypomethylation of gene RDH13 may provide a proxy or biomarker for gastric identification in ATMs.

### 3.6. Promoter Hypermethylation Represses CLDN11 Expression in Gastric ATM and CAM Group

**Figure 10:**
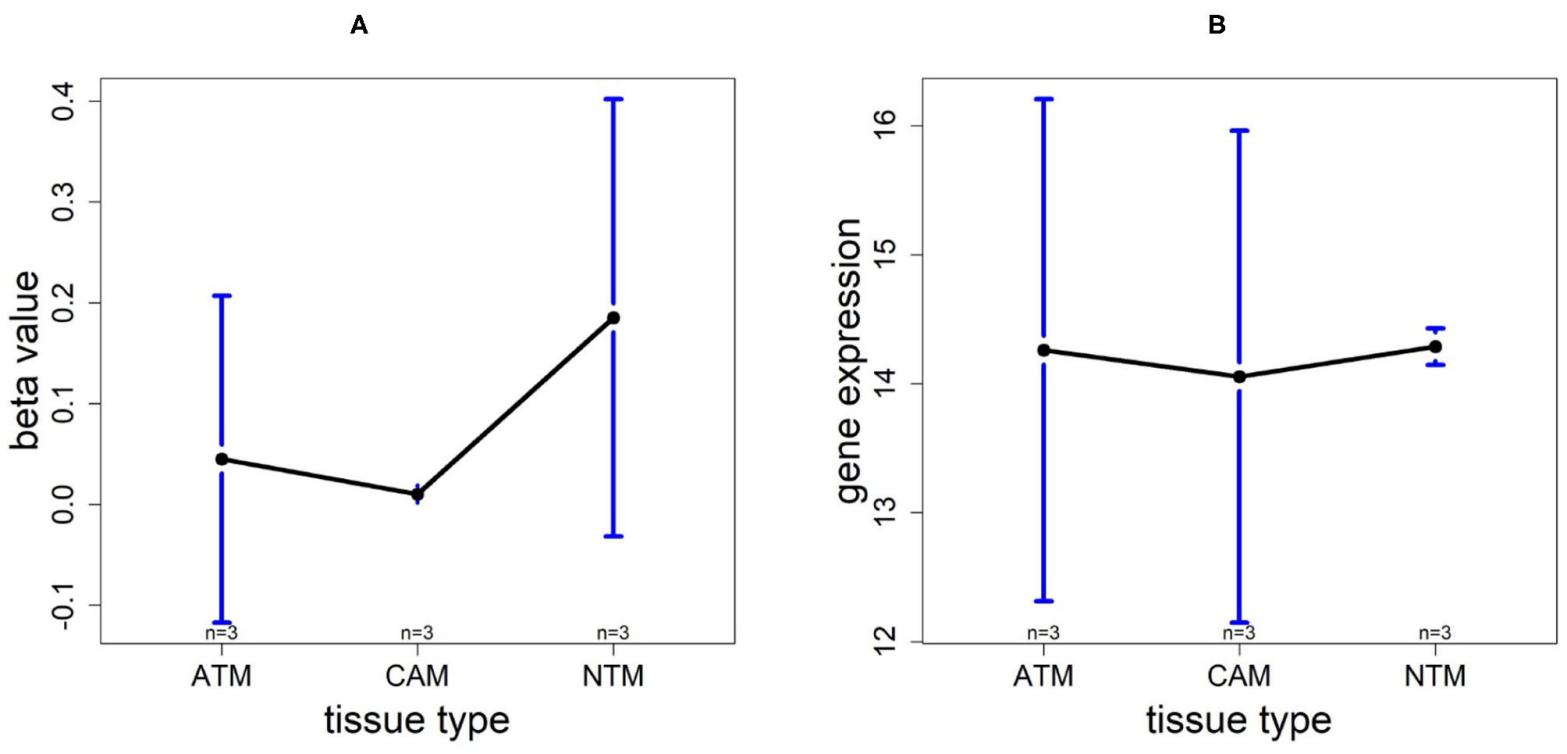
The overall methylation and gene expression levels of the CLDN11 promoter region. **(A)** Averaged methylation level over the three samples of gene CLDN11 among tissue types from GSE97686 dataset. **(B)** Averaged gene expression level over the three samples of gene CLDN11 among tissue types from GSE107161 dataset. The blue bars represent 95% confidence intervals. Some error bars are large as the sample size is only 3 in each tissue type.

Using the same procedure mentioned in section 3.5, for gene CLDN11, DNA methylation levels in each tissue type are NTM>ATM≈CAM and they are all hypomethylated. The gene expression plot indicates that CLDN11 is upregulated in ATM and CAM tissue types compared with NTMs (Figure 10). Hence, we can conclude that the hypermethylation can downregulate CLDN11 expression levels and further accelerate the development of gastric tumors. Collectively, all of the results above illustrate that CLDN11 expression may be repressed by hypermethylation in promoter region, which induces the gastric cancer.

### 3.7. Promoter Hypermethylation Represses TMTC1 Expression in Non-cancer Group

**Figure 11:**
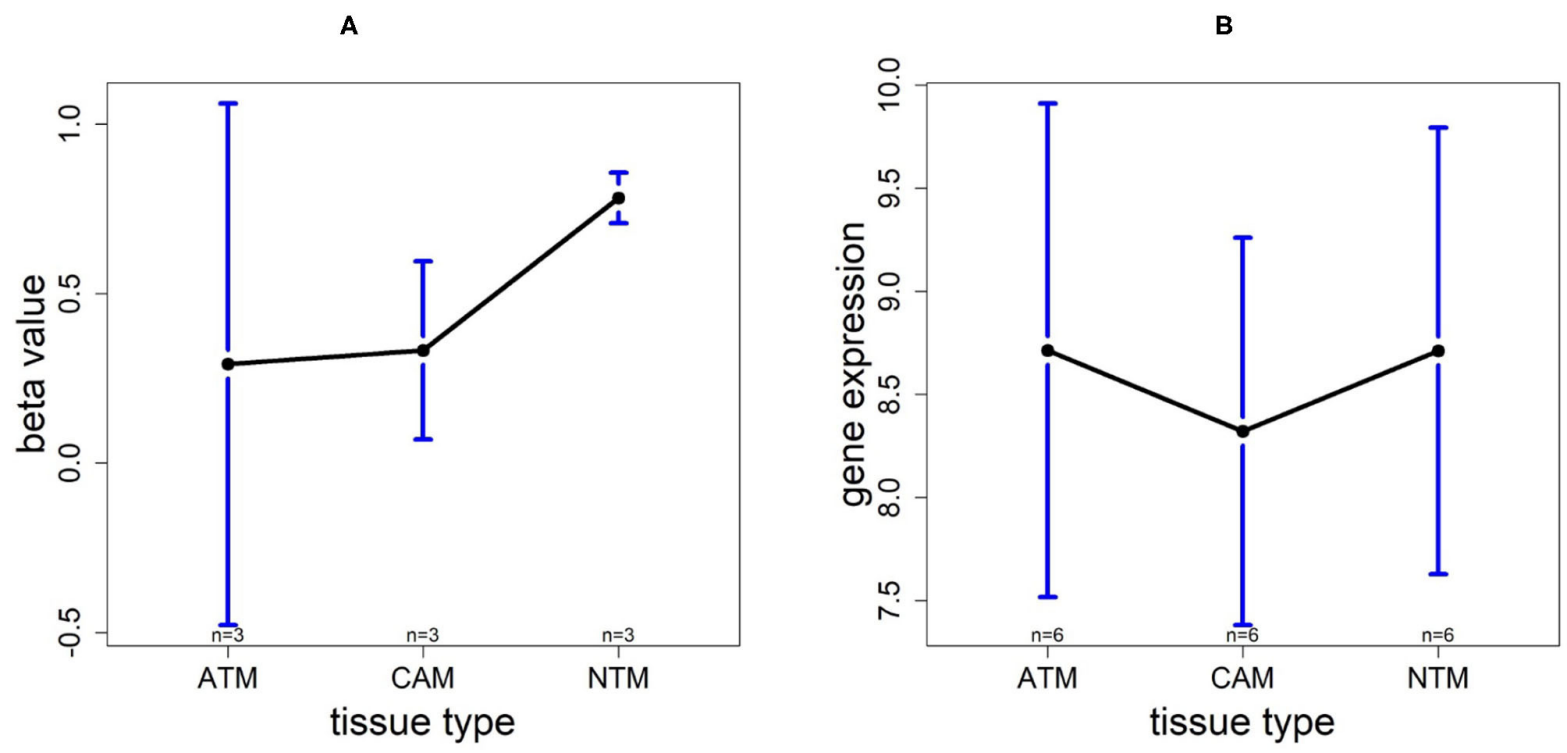
The overall methylation and gene expression levels of the TMTC1 promoter region. **(A)** Averaged methylation level over the three samples of gene TMTC1 among tissue types from GSE97686 dataset. **(B)** Averaged gene expression level over the three samples of gene TMTC1 among tissue types from GSE107161 dataset. The blue bars represent 95% confidence intervals. Some error bars are large as the sample size is only 3 in each tissue type.

In order to investigate the cancer-induced change in TMTC1 expression, as the previous two genes, we extracted the survival data of TMTC1 using TCGA data, and the gene expression data of nine samples in GSE107161 across the three tissue types. These data indicate that the level of TMTC1 promoter DNA methylation gradually changes in gastric cancer with low level in ATM and CAM tissue types and high level in NTM group which its β value is about 0.8, hypermethylation, but TMTC1 expression is almost the same among three tissue types in Figure 11. Combined with survival curve in Figure 6, we can imply that TMTC1 expression is repressed in non-cancer group, further prove TMTC1 promoter hypermethylation in NTMs will repress gene expression.

### 3.8. Promoter Hypomethylation Induces UCHL1 Expression in Gastric Cancer Group

**Figure 12:**
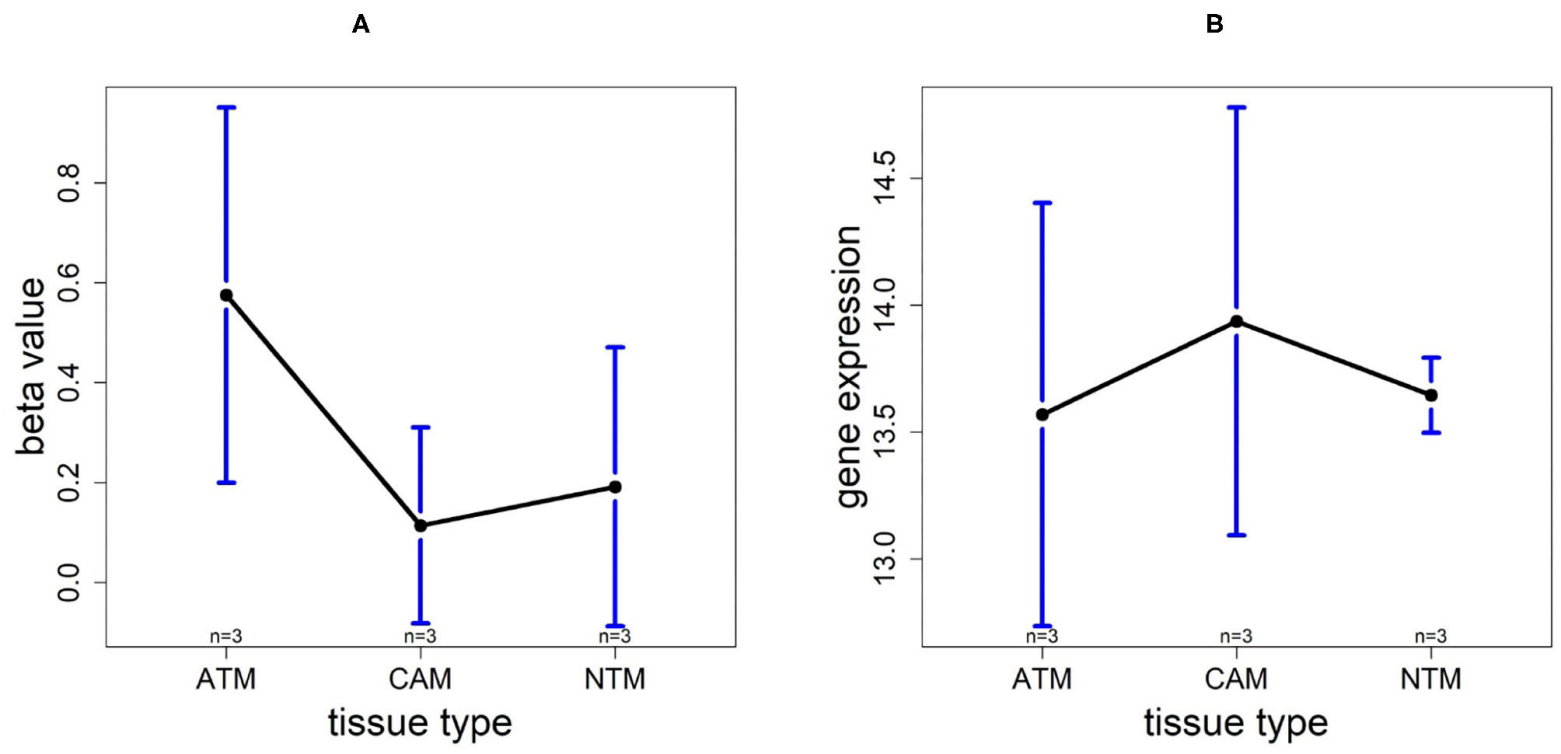
The overall methylation and gene expression levels of the UCHL1 promoter region. **(A)** Averaged methylation level over the three samples of gene UCHL1 among tissue types from GSE97686 dataset. **(B)** Averaged gene expression level over the three samples of gene UCHL1 among tissue types from GSE107161 dataset. The blue bars represent 95% confidence intervals. Some error bars are large as the sample size is only 3 in each tissue type.

Based on 9 samples, the plots of means of beta-value and gene expression among three groups confirm that these methylation trends represent a negative correlation with UCHL1 expression patterns (Figure 12). Especially, UCHL1 is significantly upregulated in CAMs, the tissue group of gastric cancers. Moreover, patients with low UCHL1 expression have longer survival time than patients with high expression (Figure 7). Therefore, We can conclude that promoter UCHL1 hypomethylation is associated with high expression level of UCHL1 expression in CAMs.

### 3.9. Promoter Hypomethylation Induces FOXP2 Expression in Non-cancer Group

**Figure 13:**
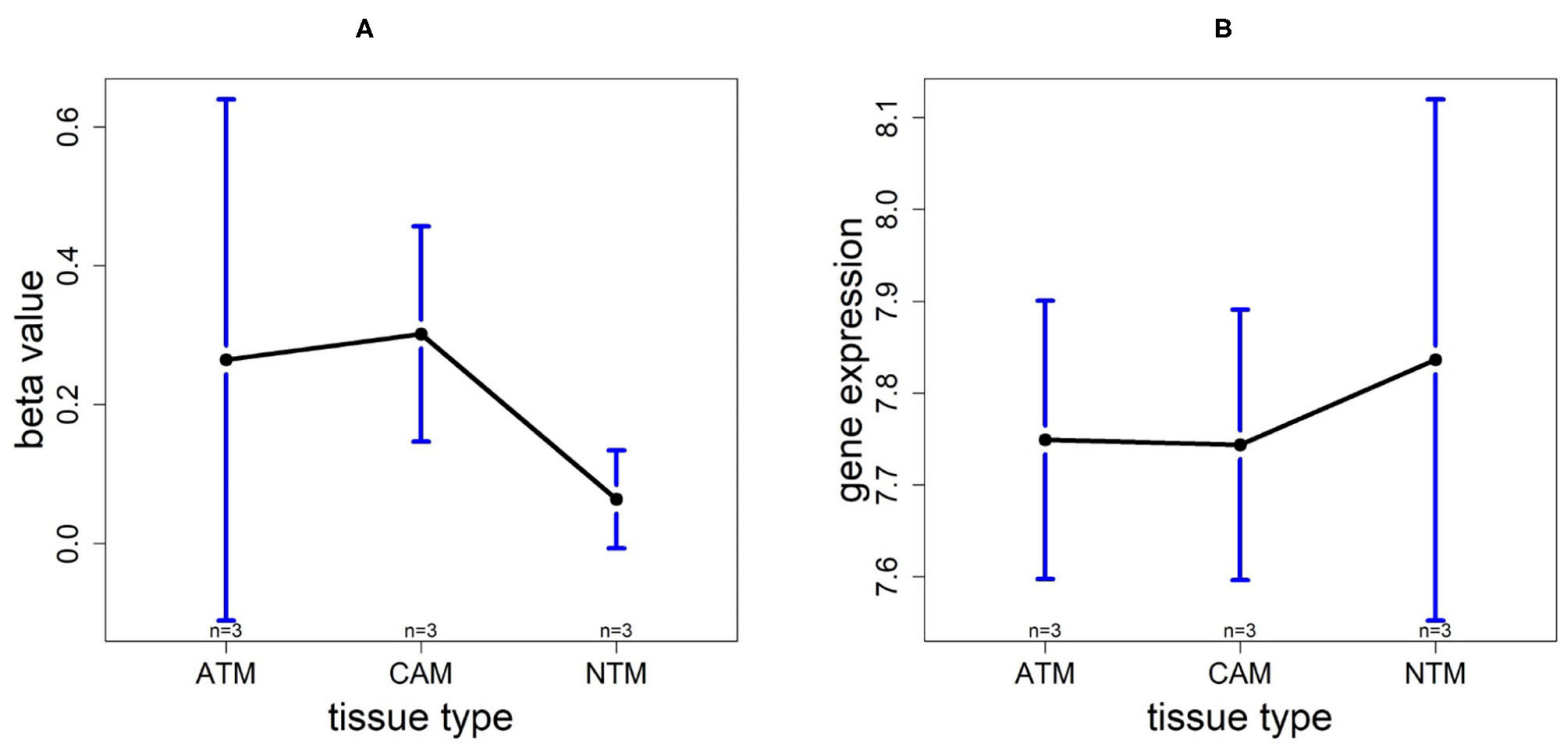
The overall methylation and gene expression levels of the FOXP2 promoter region. **(A)** Averaged methylation level over the three samples of gene FOXP2 among tissue types from GSE97686 dataset. **(B)** Averaged gene expression level over the three samples of gene FOXP2 among tissue types from GSE107161 dataset. The blue bars represent 95% confidence intervals. Some error bars are large as the sample size is only 3 in each tissue type.

For the gene FOXP2, we found that the methylation levels in ATM and CAM samples are quite similar while the level in NTMs is lower. However, FOXP2 expression level is upregulated in NTMs compared with other two tissue types (Figure 13). Thus, the upregulation of FOXP2 expression in NTMs and confirms that hypomethylation have a significantly positive effect on FOXP2 expression in non-cancer group.

## 4. Conclusion

To demonstrate the utility of BACkPAy, we chose gastric datasets containing very small sample sizes. It was shown that the Bayesian hierarchical clustering approach in BACkPAy is advantageous for data with high-dimensional but very small sample sizes. In this paper, we identified 181 differential probes that belong to eight distinct patterns. On the other hand, comparing male to female samples, we conclude that the probes within UpUp-UpDown, UpDown-UpUp, DownUp-DownDown, and DownDown-DownUp patterns have significant difference between female and male samples in NTM group, whereas, the probes from UpUp-DownUp, UpDown-DownDown, DownUp-UpUp, and DownDown-UpDown patterns have differential methylation level comparing male to female samples in CAM group. The result of patterns was visualized in 3D plots (Figure 2).

We utilized BACkPAy and LIMMA to detect probes with differential methylation levels in gastric cancer. Unlike LIMMA, BACkPAy can filter differential significant probes using Bayesian *q*-values for downstream analysis, and separate them into different biological interpretable pre-defined groups. Moreover, BACkPAy achieved satisfactory results and other current methods can not be applied when the sample size is one in each experimental group.

Further, we identified 5 prognostic genes (RDH13, CLDN11, TMTC1, UCHL1, and FOXP2) in gastric cancer. our analysis implied that DNA hypomethylation may lead to induce the RDH13 and UCHL1 expression in gastric cancer, whereas DNA hypermethylation is able to cause decreasing expression of CLDN11 in gastric groups. Promoter hypermethylation will repress the expression of TMTC1 in non-cancer group. On the contrary, Promoter hypomethylation will induce FOXP2 expression in normal tissues.

## Data Availability Statement

The datasets used and/or analyzed during the current study are available in GEO repository with accession number GSE97686 (https://www.ncbi.nlm.nih.gov/geo/query/acc.cgi?acc=GSE97686) and GSE107161 (https://www.ncbi.nlm.nih.gov/geo/query/acc.cgi?acc=GSE107161) and in Genomic Data Commons (GDC) Data Portal with project ID TCGA-STAD (https://portal.gdc.cancer.gov/).

## Author Contributions

TC: conceptualization. TC and XC: methodology. XC: data extraction and implementation of the methods and writing of the first draft. TC, XC, and QZ: formal analysis, investigation and writing and editing. All authors read and approved the final manuscript.

## Conflict of Interest

The authors declare that the research was conducted in the absence of any commercial or financial relationships that could be construed as a potential conflict of interest.
