# Supplementary material for: Filtering High-Dimensional Methylation Marks With Extremely Small Sample Size: An Application to Gastric Cancer Data
Source: Front Genet. 2021 Jul 12;12:705708. doi: 10.3389/fgene.2021.705708 (PMC8313381; doi:10.3389/fgene.2021.705708)
Supplement: Supplementary file 1 [file Data_Sheet_1.PDF]

Supplementary Material for "Filtering high-dimensional methylation marks with extremely small sample size: an application to gastric cancer data"

1 SUPPLEMENTARY TABLE

Table S1: The result of significant genes with TCGA data by Cox model

	probe	gene name	pattern	p-value	adjusted p-value
1	cg18743287	RDH13	UpUp-UpDown	6e-04	0.0325
2	cg17078427	CLDN11	UpUp-DownUp	8e-04	0.0325
3	cg05471616	TMTC1	DownUp-UpUp	0.001	0.0325
4	cg09921610	UCHL1	DownUp-DownDown	0.001	0.0325
5	cg20050108	FOXP2	UpDown-DownDown	0.0013	0.0338
6	cg12640049	IGSF21	DownDown-DownUp	0.0024	0.052
7	cg22026089	PDZRN4	UpDown-DownDown	0.005	0.065
8	cg23282441	CDH23	UpDown-DownDown	0.0046	0.065
9	cg00399374	CHMP4C	DownUp-DownDown	0.0035	0.065
10	cg23496906	UBE2E1	DownDown-UpDown	0.0049	0.065
11	cg27121538	SV2B	DownUp-DownDown	0.006	0.0672
12	cg15609631	FAM108C1	DownDown-UpDown	0.0062	0.0672
13	cg15444648	FLJ42709	UpUp-DownUp	0.0095	0.0823
14	cg16600634	FLJ42709	UpUp-DownUp	0.0095	0.0823
15	cg25400864	INTS2	DownUp-DownDown	0.0091	0.0823
16	cg17519938	LRRC47	UpUp-UpDown	0.0118	0.0948
17	cg01173941	TPK1	DownUp-DownDown	0.0124	0.0948
18	cg02311932	RBM24	DownUp-UpUp	0.0133	0.0961
19	cg22367705	SLC9A1	UpDown-DownDown	0.0166	0.1124
20	cg02159271	ZNF76	DownUp-UpUp	0.0173	0.1124
21	cg05551114	C1QTNF7	DownUp-UpUp	0.0184	0.1129
22	cg10103850	SLC8A1	DownUp-UpUp	0.0191	0.1129
23	cg05284727	WHSC1	DownUp-UpUp	0.02	0.113
24	cg00919857	LYSMD2	DownUp-UpUp	0.0243	0.1316
25	cg19284039	ASCL2	DownUp-UpUp	0.0276	0.1435
26	cg06870118	TTLL3	UpDown-DownDown	0.0297	0.1485
27	cg02126435	RFWD3	UpDown-DownDown	0.0368	0.1772
28	cg03830585	ITPR1	DownUp-UpUp	0.0455	0.208
29	cg00919857	TMOD2	DownUp-UpUp	0.0464	0.208
30	cg10842095	TIMELESS	UpUp-DownUp	0.0589	0.2494
31	cg22465736	SPAG5	DownUp-UpUp	0.0614	0.2494
32	cg12017722	ISG20	DownUp-UpUp	0.0614	0.2494

33	cg11462533	HPCAL4	DownUp-DownDown	0.0725	0.2856
34	cg00296121	FRMD4A	DownUp-DownDown	0.0817	0.295
35	cg25453664	FRMD4A	DownUp-DownDown	0.0817	0.295
36	cg05700142	TAPBP	DownUp-DownDown	0.0774	0.295
37	cg01812146	ARHGAP29	UpDown-UpUp	0.0846	0.2972
38	cg01781446	ZBTB4	UpDown-UpUp	0.0985	0.3148
39	cg02633817	FXYD3	UpDown-DownDown	0.1	0.3148
40	cg27598340	EGFR	UpDown-DownDown	0.1011	0.3148
41	cg26588194	TIGD6	DownUp-UpUp	0.1017	0.3148
42	cg04661674	GABBR1	DownUp-DownDown	0.0952	0.3148
43	cg17861481	GNAI3	UpUp-UpDown	0.1128	0.3259
44	cg07036530	GPR26	DownUp-UpUp	0.1126	0.3259
45	cg11777611	RGS9	DownDown-DownUp	0.1092	0.3259
46	cg07895203	PALLD	DownUp-UpUp	0.1371	0.3875
47	cg00584422	ERN2	DownUp-DownDown	0.1537	0.4251
48	cg01658502	RUFY4	UpDown-DownDown	0.1633	0.4423
49	cg15227911	CHD3	UpDown-DownDown	0.178	0.455
50	cg26888063	EDAR	DownUp-DownDown	0.1759	0.455
51	cg25636933	CEP135	DownDown-UpDown	0.1785	0.455
52	cg19678392	PON1	UpDown-DownDown	0.1878	0.4606
53	cg26661922	WWP2	DownUp-DownDown	0.1862	0.4606
54	cg26853855	CSRNP3	UpDown-UpUp	0.2032	0.4803
55	cg06457011	PLCG1	DownUp-DownDown	0.2023	0.4803
56	cg07916022	GPR39	UpUp-DownUp	0.2243	0.486
57	cg12816057	HTR4	UpUp-DownUp	0.2134	0.486
58	cg15833099	GPR39	UpUp-DownUp	0.2243	0.486
59	cg27048432	GPR39	UpUp-DownUp	0.2243	0.486
60	cg23282441	C10orf54	UpDown-DownDown	0.2095	0.486
61	cg23930923	ZNF665	DownUp-UpUp	0.2731	0.5598
62	cg16704590	SNORD12B	DownUp-UpUp	0.2712	0.5598
63	cg01794265	FAM105A	DownUp-DownDown	0.2706	0.5598
64	cg21452411	PRSS22	DownUp-DownDown	0.2756	0.5598
65	cg23330451	PRR23B	DownUp-UpUp	0.2932	0.5775
66	cg18024167	HAPLN1	DownDown-UpDown	0.2925	0.5775
67	cg00101715	FAM55C	DownUp-UpUp	0.3054	0.5792
68	cg13356175	S100A7L2	DownUp-UpUp	0.3034	0.5792
69	cg10306247	ZNF192	DownUp-UpUp	0.3074	0.5792
70	cg19361269	CPLX4	UpUp-UpDown	0.3322	0.6043
71	cg08894487	ARID3A	UpDown-UpUp	0.3347	0.6043
72	cg15204036	AGXT2L1	DownUp-UpUp	0.329	0.6043
73	cg20269537	ATXN10	DownUp-DownDown	0.3485	0.6206
74	cg06267075	TMCO7	DownDown-DownUp	0.3846	0.6756
75	cg02367655	LOC100302401	UpDown-DownDown	0.427	0.739
76	cg23727043	ADAMTS7	DownUp-UpUp	0.4391	0.739
77	cg17959549	PXT1	DownUp-UpUp	0.4408	0.739

78	cg12991093	NNT	DownUp-DownDown	0.4434	0.739
79	cg22898362	KLK9	UpDown-UpUp	0.461	0.7404
80	cg03541338	RGS10	UpDown-DownDown	0.467	0.7404
81	cg16704590	SNORD12	DownUp-UpUp	0.4585	0.7404
82	cg15878685	UGT2A3	DownDown-UpDown	0.465	0.7404
83	cg07916022	LYPD1	UpUp-DownUp	0.4861	0.7434
84	cg15833099	LYPD1	UpUp-DownUp	0.4861	0.7434
85	cg27048432	LYPD1	UpUp-DownUp	0.4861	0.7434
86	cg22892043	BEST1	UpDown-UpUp	0.5123	0.7445
87	cg13261623	AANAT	DownUp-DownDown	0.5123	0.7445
88	cg04119462	ABCA17P	DownUp-DownDown	0.5087	0.7445
89	cg24142633	C17orf67	DownDown-UpDown	0.5154	0.7445
90	cg25332826	C17orf67	DownDown-UpDown	0.5154	0.7445
91	cg05474128	DOCK1	UpUp-DownUp	0.5495	0.7663
92	cg22898362	KLK8	UpDown-UpUp	0.5523	0.7663
93	cg01261503	POLG2	UpDown-DownDown	0.5484	0.7663
94	cg17959549	KCTD20	DownUp-UpUp	0.56	0.7663
95	cg03957898	FLG2	DownDown-UpDown	0.5595	0.7663
96	cg01523769	ANKRD11	UpDown-DownDown	0.5778	0.7744
97	cg00158308	OXGR1	DownUp-UpUp	0.5769	0.7744
98	cg23876131	TRRAP	UpDown-UpUp	0.601	0.7892
99	cg16021018	CCRL2	DownDown-UpDown	0.5971	0.7892
100	cg05377120	KLRD1	DownDown-DownUp	0.6093	0.7921
101	cg02014107	DLX2	DownUp-DownDown	0.6191	0.7969
102	cg26588194	HMGXB3	DownUp-UpUp	0.6337	0.8002
103	cg19921355	PODNL1	DownUp-DownDown	0.6402	0.8002
104	cg14218233	GALNT2	DownUp-DownDown	0.6361	0.8002
105	cg16153547	LBX2	UpUp-DownUp	0.6522	0.8066
106	cg22892043	FTH1	UpDown-UpUp	0.6586	0.8066
107	cg26643047	ATP6V1E2	UpDown-DownDown	0.6701	0.8066
108	cg02766173	PPP2R2C	DownUp-DownDown	0.6645	0.8066
109	cg14077898	SLC17A8	DownUp-DownDown	0.6811	0.8123
110	cg19552640	MEGF10	UpDown-UpUp	0.6951	0.8141
111	cg02691325	TMEM151B	DownUp-UpUp	0.6913	0.8141
112	cg08739188	SV2C	UpDown-DownDown	0.7368	0.8476
113	cg10919522	C14orf43	DownUp-UpUp	0.7353	0.8476
114	cg07303450	DOC2A	UpDown-UpUp	0.7736	0.8523
115	cg09241332	ZIC1	UpDown-UpUp	0.7597	0.8523
116	cg17129400	NCOA6	DownUp-UpUp	0.7673	0.8523
117	cg13433363	KCNK15	DownUp-DownDown	0.7674	0.8523
118	cg16836028	MYH9	DownDown-UpDown	0.7495	0.8523
119	cg07916058	XPR1	UpUp-UpDown	0.7887	0.8616
120	cg23891049	BPGM	UpUp-DownUp	0.8745	0.926
121	cg00704970	RPTOR	DownUp-UpUp	0.8761	0.926
122	cg06763161	PHC2	DownUp-DownDown	0.8651	0.926

123	cg14258756	KCNAB2	DownDown-UpDown	0.8602	0.926
124	cg08325929	GABRG3	DownUp-UpUp	0.8904	0.9335
125	cg18554116	TRAPPC9	UpDown-DownDown	0.9238	0.9608
126	cg20899581	HIST1H3I	DownUp-UpUp	0.9431	0.973
127	cg02367655	RASAL2	UpDown-DownDown	0.9574	0.98
128	cg20899581	HIST1H4L	DownUp-UpUp	0.9726	0.9878
129	cg02423044	KLF14	UpDown-UpUp	0.9999	0.9999
130	cg10169539	NIN	UpDown-DownDown	0.9923	0.9999

Note: Each color in the table represents one specific pattern genes belong to. e.g. genes in blue color belong to the pattern UpUp-UpDown and genes in black color belong to the pattern DownUp-UpUp.

2 FDR CONTROL

The false discovery rate (FDR) is a statistical approach used in multiple hypothesis testing to correct for multiple comparisons. Benjamini and Hochberg (1995) uses this formula as FDR

$$FDR = E[V/R \mid R > 0]P(R > 0) \tag{S1}$$

where V is the number of false discoveries, R is the number of discoveries i.e. rejections of the null hypothesis and FDR is the expected value of proportions of false discoveries (V) among all discoveries (R). To control FDR at level α, the Benjamini–Hochberg procedure (BH step-up procedure) works as follows (Benjamini and Hochberg, 1995): Suppose that the p-values resulting from m tests are ordered that $p_{(1)} \leq p_{(2)} \leq \cdots \leq p_{(m)}$. If we calculate

$$\hat{k} = \arg \max_{1 \leq k \leq m} \{k : p_{(k)} \leq \alpha \cdot k/m\} \tag{S2}$$

then rejecting the null hypotheses corresponding to $p_{(1)}, \cdots, p_{(\hat{k})}$ provides FDR = $m0/m \cdot \alpha \leq \alpha$, where m_0 is the number of all true null hypotheses. If no p-value satisfies this inequality, then no hypothesis test is called significant. Storey (2003) introduced a modified version of the FDR that allows to define *q-values* which is a natural Bayesian posterior p-value. BACkPAy uses q-values to detect differential features.

3 COX REGRESSION MODEL

The Cox model is expressed by the hazard function denoted by $h(t)$. Briefly, the hazard function can be interpreted as the risk of dying at time t (Bradburn et al., 2003). In our study, it can be estimated as follow

$$h(t) = h_0(t) \exp\{b_1 X_1\} \tag{S3}$$

where t is the survival time, $h(t)$ represents the estimated hazard function determined by variable X_1 (e.g, gene expression level), coefficient b_1 measures the impact of variable X_1 and $h_0(t)$ is baseline hazard corresponding to the hazard if $X_1 = 0$. Based on the hazard function, the survival function can also be derived, which represents the probability that a subject can survive beyond time T. The formula is

$$S(t) = \exp\left\{-\int_0^t h(s)ds\right\}. \tag{S4}$$

REFERENCES

Benjamini, Y. and Hochberg, Y. (1995). Controlling the false discovery rate: a practical and powerful approach to multiple hypothesis testing. *Journal of the Royal Statistical Society* 57, 289–300. doi:www.jstor.org/stable/2346101

Bradburn, M. J., Clark, T. G., Love, S. B., and Altman, D. G. (2003). Survival analysis part ii: Multivariate data analysis – an introduction to concepts and methods. *British Journal of Cancer* 89, 431–436. doi:https://doi.org/10.1038/sj.bjc.6601119

Storey, J. D. (2003). The positive false discovery rate: A bayesian interpretation and the q-value. *The Annals of Statistics* 31, 2013–2035. doi:10.1214/aos/1074290335
